# Immune Aging Within the Tumor Microenvironment Predicts Survival in Lung Adenocarcinoma

**DOI:** 10.3390/cancers18091343

**Published:** 2026-04-23

**Authors:** Taeyun Kim, Hyunji Choi, Tae Won Jang, Chul-Ho Oak

**Affiliations:** 1 Division of Pulmonology, Department of Internal Medicine, Kosin University College of Medicine, Kosin University Gospel Hospital, Busan 49267, Republic of Korea; 2 Department of Laboratory Medicine, Kosin University College of Medicine, Kosin University Gospel Hospital, Busan 49267, Republic of Korea

**Keywords:** tumor microenvironment, NSCLC, lung adenocarcinoma, immunosenescence, prognosis

## Abstract

**Background/Objectives**: Immune aging has been associated with survival outcomes in patients with lung adenocarcinoma (LUAD), but its relevance within the tumor microenvironment (TME) remains unclear. **Methods**: Clinical, RNA-sequencing, and somatic mutation data from the TCGA LUAD cohort were analyzed. Immune aging score within the TME was quantified using a predefined blood-driven 121-gene immune aging signature (IAS-121), and patients were categorized into the lowest versus the highest IAS-121 tertiles. Immune cell composition in the TME was inferred using xCell. Overall survival (OS) was evaluated using Kaplan–Meier analysis, Cox proportional hazards models adjusted for age, sex, tumor stage, smoking status, and EGFR mutation status, and restricted cubic spline analysis to examine the dose–response relationship between IAS-121 and mortality risk. Sensitivity analyses comparing the highest versus lowest quartiles or higher than median versus lower than median of IAS-121 were performed. Two independent LUAD cohorts (GSE68465 and GSE50081) were employed for validation. **Results**: A total of 518 patients with LUAD from the TCGA cohort were analyzed. Restricted cubic spline analysis showed a linear association between IAS-121 and OS. Patients in the highest IAS-121 tertile showed significantly better survival than those in the lowest tertile in both the TCGA cohort (*p* < 0.001) and the external validation cohorts (*p* = 0.003). In multivariable-adjusted Cox models, the lowest IAS-121 tertile was associated with worse survival in TCGA (adjusted HR 1.87, 95% CI 1.20–2.92) and in the pooled external cohorts (adjusted HR 1.57, 95% CI 1.02–2.43). Subgroup analyses showed generally consistent associations across clinical strata. Tumors with higher IAS-121 exhibited lower CD8^+^ and CD4^+^ naïve T-cell enrichment but higher neutrophil infiltration. **Conclusions**: Immune aging within TME is associated with poorer survival in LUAD. Given this study is hypothesis-generating, further investigations integrating tissue- and blood-based measures of immune aging are warranted to clarify its clinical and biological implications.

## Simple Summary

Immune aging has been linked to adverse clinical outcomes in various diseases, but its role within the tumor microenvironment (TME) of lung adenocarcinoma (LUAD) remains unclear. In this study, we applied a validated 121-gene immune aging signature, originally derived from peripheral blood, to tumor transcriptomic data. We found that higher immune aging within the TME was associated with distinct immunologic features, including reduced T-cell–mediated activity and increased myeloid-driven inflammation. Importantly, patients with higher tumor immune aging showed significantly worse overall survival, independent of conventional clinical factors. These findings suggest that immune aging within the TME reflects biologically meaningful immune dysfunction beyond chronological age and may serve as a novel prognostic biomarker in LUAD.

## 1. Introduction

Non–small cell lung cancer (NSCLC) remains the leading cause of cancer-related death worldwide, and survival outcomes vary widely even among patients with similar clinical and genomic profiles [1]. In recent years, increasing attention has focused on the tumor microenvironment (TME) as a critical determinant of tumor progression, immune evasion, and response to therapies [2]. The TME comprises diverse immune and stromal cell populations whose composition and functional states dynamically interact with tumor cells [3]; thus, characterizing the immunologic landscape within the TME has become essential for understanding heterogeneity in clinical outcomes in patients with NSCLC [4,5].

Aging is accompanied by a progressive decline in immune competence—termed immune aging (immunosenescence)—which includes reduced naïve T-cell pools, expansion of terminally differentiated effector cells, impaired cytokine signaling, and chronic low-grade inflammation [6]. However, immune aging does not necessarily correlate with chronological age. For example, a recent longitudinal multi-omics study established a clinically meaningful metric of immune age derived from immune profiling of healthy adults over nine years [7]. In this study, immune age was heterogeneous across individuals and predicted all-cause cardiovascular mortality more robustly than epigenetic aging markers.

In NSCLC, particularly in the most common subtype, lung adenocarcinoma (LUAD), evidence about the link between immune aging and clinical outcomes remains limited [8,9]. Although older patients may show altered responses to immune checkpoint inhibitors (ICIs) [10], such findings cannot be extrapolated to the biological effects of immune aging within tumors [7]. Traditional age-related assessments—such as performance status [11] or geriatric scoring [12]—capture functional decline but do not reflect the molecular alterations underlying immune aging. Given that immune cells within TME undergo chronic antigenic stimulation, exhaustion, and phenotypic remodeling [13], the assessment of immune aging within the TME would more accurately capture the local anti-tumor immune capacity than chronological age or systemic immune markers.

In this context, the present study aims to evaluate the prognostic significance of TME-specific immune aging in LUAD using a validated immune aging gene-expression signature derived from prior multi-omic analyses. By quantifying immune aging within tumor tissue, this study was conducted to investigate whether TME immune age provides prognostic value in NSCLC LUAD.

## 2. Materials and Methods

### 2.1. Data Source and Study Participants

We performed a retrospective analysis of patients with NSCLC included in The Cancer Genome Atlas (TCGA) LUAD cohorts. Clinical, RNA-sequencing, and somatic mutation data were obtained from the Genomic Data Commons (GDC) portal using the *TCGAbiolinks* R package. For each project, we queried tumor samples with “Transcriptome Profiling—Gene Expression Quantification—STAR—Counts” workflow and “Simple Nucleotide Variation—Masked Somatic Mutation” files, together with curated clinical data.

For clinical information, we collected age, sex, tumor stage, smoking status, and the presence of EGFR mutation. Tumor stage was recategorized into three groups: stage I–II, stage III, and Stage IV. Smoking status was categorized into never, former, and current smokers. Age was dichotomized into <65 versus ≥65 years. We also collected the presence of EGFR mutation (mutated versus wild type). Overall survival (OS) was defined as time from the TCGA index date to death or last follow-up, calculated as the number of days to death or, if unavailable, days to last follow-up. OS time was converted to months.

### 2.2. Immune Aging Score (IAS)-121 Calculation

#### 2.2.1. RNA-Seq Data Preprocessing

Raw gene-level count matrices were normalized and transformed using a standard pipeline: *Ensembl* gene identifiers were mapped to HUGO Gene Nomenclature Committee gene symbols. Then, library size normalization and log2-counts-per-million (logCPM) transformation were applied prior to downstream analyses.

#### 2.2.2. xCell-Derived Immune Cell Scores

To characterize TME, we applied xCell to the logCPM expression matrix to infer enrichment scores for immune cell subpopulations. Six immune cell types conceptually reflecting immune aging—CD8^+^ T cells, CD4^+^ naïve T cells, NK cells, regulatory T cells (Tregs), M2 macrophages, and neutrophils—were selected *a priori*. Selected immune cell populations were chosen based on their established roles in immunosenescence and tumor immune regulation, representing both effector and immunosuppressive components of the TME. For each tumor sample, xCell scores for these six cell types were extracted and standardized to z-scores across samples. When multiple tumor samples were available for the same patient, we aggregated to the patient level.

#### 2.2.3. IAS-121 Calculation

**Figure 1:**
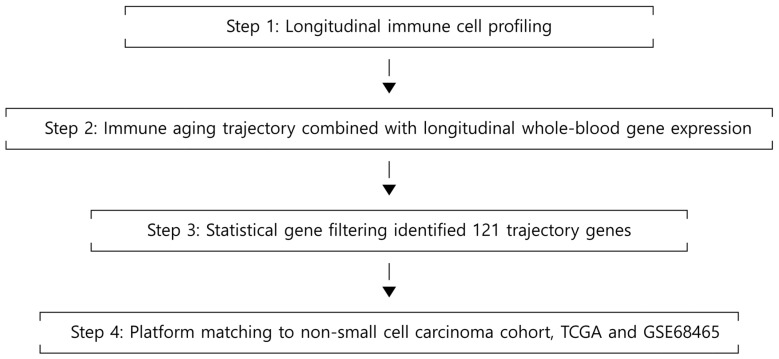
IMM-AGE modeling and its integration into the current study design.

The immune aging (IMM-AGE) score was originally developed by modeling the longitudinal trajectory of immune aging using peripheral blood samples [7]. The 121-gene transcriptional signature derived from this model comprises genes that show consistent changes across both chronological and immunologic aging trajectories [7]. We extracted this 121-gene set and mapped it to the TCGA tumor transcriptome. The schematic illustrating the IMM-AGE modeling process and its application to our study is shown in Figure 1. For each gene, expression values were standardized across samples using gene-wise z-scoring, and the published IMM-AGE direction coefficient was applied as a gene-specific weight. A sample-level IMM-AGE score was then calculated as the mean of the weighted standardized gene expression values. When multiple tumor samples were available for a single patient, sample-level scores were averaged to obtain a patient-level IAS-121 value. The resulting IAS-121 distribution was trichotomized (highest tertile vs. lowest tertile) and additionally categorized into quartiles for sensitivity analyses.

### 2.3. Statistical Analysis

All analyses were conducted in R version 4.2.1, and two-sided *p* values < 0.05 were considered statistically significant. Baseline clinicopathologic characteristics were summarized by IAS-121 tertile groups.

OS was evaluated using Kaplan–Meier curves with log-rank tests. Cox proportional hazards models were fitted to assess whether IAS-121 remained an independent prognostic factor. The proportional hazards assumption was assessed using Schoenfeld residuals, and no significant violations were detected. Model diagnostics were performed to evaluate the adequacy of the fitted models. Restricted cubic spline models were constructed to evaluate the potential non-linear association between IAS-121 and survival outcomes. Four knots were placed at the 5th, 35th, 65th, and 95th percentiles of the IAS-121 distribution, following commonly recommended practices. The models were fitted using adjusted Cox proportional hazards regression, and the linearity assumption was assessed based on the spline terms. We also employed a forest plot to identify any interaction between subgroups. Subgroups were stratified by age, sex, stage, smoking status, and EGFR mutation. Sensitivity analysis was performed by categorizing IAS-121 into quartile and median groups. HR and 95% CI were estimated. To validate the results, we performed an additional pooled analysis using other LUAD cohorts, GSE68465 [14] and GSE50081 [15].

To evaluate immunologic differences associated with tumor immune aging, xCell-derived standardized scores for several immune cell types (CD8^+^ T cells, CD4^+^ naïve T cells, NK cells, Tregs, M2 macrophages, neutrophils) were compared between IAS-121 groups using Wilcoxon rank-sum tests and visualized with boxplots.

For LUAD samples, the 121-gene signature was visualized using a heatmap. Gene-wise z-scored expression values were ordered by increasing IAS-121, with annotations for clinical variables. Hierarchical clustering was applied to genes only.

## 3. Results

A total of 518 LUAD patients in TCGA were included. Baseline characteristics by IAS-121 tertile (173 in lowest vs. 173 in highest) groups are shown in Table 1. Patients with higher IAS-121 were younger, more often male, and current smokers; smoking status and EGFR mutation were similar between groups.

**Figure 2:**
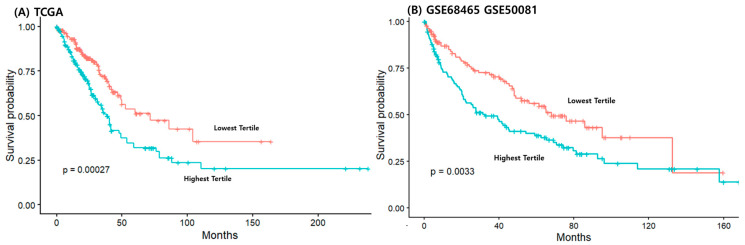
Overall survival according to immune aging score-121 tertiles in TCGA and external validation cohorts (GSE68465 and GSE50081).

Restricted cubic spline analysis showed a linear relationship between IAS-121 and OS in LUAD patients (Figure S1). The highest IAS-121 tertile group showed lower survival compared with the lowest tertile group (Figure 2A, *p* < 0.001). This was similar in another pooled analysis using two cohorts, GSE68465 and GSE50081 (Figure 2B, *p* = 0.003). A sensitivity analysis comparing patients in the lowest quartile and the highest quartile showed similar results in both TCGA and external cohorts, GSE68465 and GSE50081 (Figure S2). Another analysis based on the median value of IAS-121 was also consistent (Figure S3).

The Cox proportional hazard model showed that in the TCGA LUAD, patients in the highest tertile of the IAS-121 had significantly worse overall survival compared with those in the lowest tertile in the crude model (HR, 1.86; 95% CI, 1.33–2.62; *p* < 0.001, Table 2). This association remained significant after adjustment for age, sex, tumor stage, smoking status, and EGFR mutation status (adjusted HR, 1.87; 95% CI, 1.20–2.92; *p* = 0.006). Consistent findings were observed in the external validation cohort combining GSE68465 and GSE50081, where the highest tertile was associated with poorer survival in both the crude model (HR, 1.68; 95% CI, 1.19–2.39; *p* = 0.004) and the multivariable-adjusted model (adjusted HR, 1.57; 95% CI, 1.02–2.43; *p* = 0.007).

The forest plot showed generally consistent subgroup interaction across age, sex, tumor stage, smoking status, and EGFR mutation, although the statistical significance was only obtained in LUAD (Figure S4).

**Figure 3:**
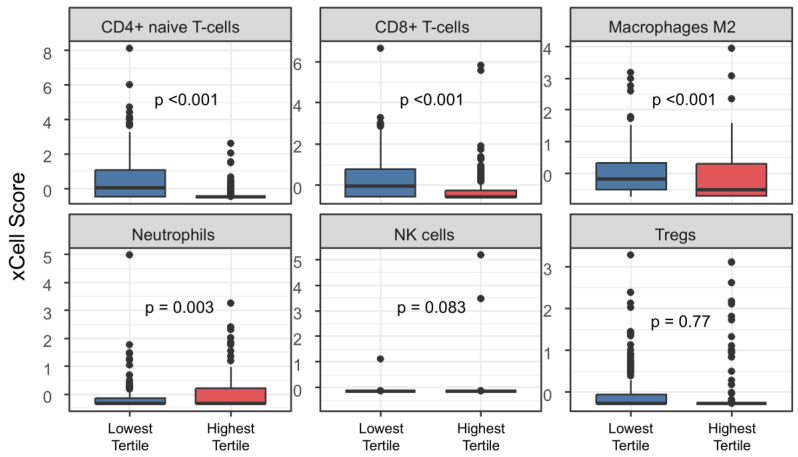
Immune cell enrichment score in LUAD according to IAS-121.

Higher IAS-121 was associated with a distinct TME characterized by lower CD8^+^ T-cell, CD4^+^ naïve T-cell, and macrophage enrichment (Figure 3). Whereas, in patients with high IAS-121, neutrophil enrichment scores were higher than in patients with low IAS-121 scores.

To examine transcriptional patterns underlying tumor immune aging, we constructed a heatmap of the 121 immune aging-related genes in the LUAD sample (Figure S5). Although the discriminative pattern was not visually significant, samples ordered by increasing IAS-121 showed a coordinated shift in gene expression across the signature. Clinical annotations showed no clustering driven by stage, smoking status, or EGFR mutation.

## 4. Discussion

This study is not confirmatory, but rather hypothesis-generating. Immune aging is an important determinant of survival in patients with LUAD. In this study, we applied a validated blood sample-driven immune-aging gene signature to tumor tissue samples to evaluate the prognostic role of immune aging in LUAD. Our study is the first to show that immune aging within the TME also carries significant prognostic relevance in patients with LUAD. Using applied tumor transcriptomes in TCGA, GSE68465, and GSE50081 cohorts, we found that higher intra-tumoral immune aging was associated with reduced lymphocytic activity, increased myeloid-inflammatory features, and poorer OS. These associations were consistent across multiple subgroup analyses, highlighting the robustness of TME-specific immune aging as a prognostic factor. Together, our findings suggest that tumor immune aging captures biologic dysfunction not merely reflected by chronological age alone and may offer a useful framework for risk stratification in LUAD.

One of the main findings is that immune aging as a determinant of survival in LUAD is not confined to systemic or peripheral blood measures but also manifests within the TME. Prior research has mainly characterized immune aging through circulating immune profiles [10,16,17,18], which could overlook the cumulative antigenic stimulation, exhaustion, and remodeling that occur locally within tumors. However, recent studies on TME consistently show that local immune dynamics are strongly shaped by persistent antigen exposure, metabolic stress, and immunosuppressive signaling, leading to distinct immune phenotypes that are not fully reflected in peripheral blood [19,20]. By applying a validated immune-aging gene signature to tumor tissue, intra-tumoral immune aging does capture distinct immunologic alterations linked to poorer clinical outcomes. We observed that higher TME immune aging was characterized by increased myeloid-cell signatures and reduced T-cell activity, reflecting a shift toward a more suppressive and less cytotoxic immune landscape. This pattern is consistent with blood-based findings showing that elevated neutrophil–lymphocyte ratios (NLRs) are associated with worse survival in NSCLC [21,22]. A high NLR reflects increased neutrophil-driven inflammation and reduced lymphocyte-mediated anti-tumor immunity. This imbalance promotes an immunosuppressive environment that facilitates tumor progression, explaining its association with poor survival. Treatment response to ICI was lower in NSCLC patients with higher baseline NLR [22].

In this study, we used data from tissue samples derived from the TCGA dataset. To account for immune aging within NSCLC tumor tissue, we employed previously confirmed gene signatures related to aging. The 121-gene signature was originally developed as an RNA-based approximation of systemic immune age rather than as a metric of intratumoral immunity. In contrast, TCGA bulk RNA-seq reflects transcriptional programs of tumor-infiltrating immune cells and surrounding stromal and malignant components, which represent a biologically distinct context. Consequently, the IAS-121 score used in our analysis should not be interpreted as a direct surrogate of systemic immune age; instead, it reflects a tumor immune aging–like transcriptional program, characterized by shifts toward terminal differentiation, cytotoxic/exhausted phenotypes, and reduced naïve-like signatures within the tumor microenvironment. Tumor-infiltrating lymphocytes are recruited from the circulating immune pool through chemokine-mediated trafficking from peripheral blood into tumor sites, and their differentiation and exhaustion states mirror systemic, age-related remodeling of T-cell immunity observed in cancer patients [23,24,25]. Therefore, a blood-derived immunosenescence signature may still capture the extent to which intratumoral immune cells manifest systemic immune aging within the TME. In a clinical context, recent studies in this field have robustly proven a clear association between peripheral gene signature and immune profiles within TME of breast [26] and head and neck cancer [27]. However, such associations do not imply direct biological equivalence. Therefore, further study is needed to clarify how this tumor-derived immune-aging relates to systemic immune aging and to its clinical relevance in LUAD.

It is well known that the immune landscape is different between LUAD and LUSC [28]. LUAD may be more sensitive to immune-aging–related alterations because its TME generally exhibits greater variability in lymphocytic infiltration and myeloid-driven inflammation than LUSC [28,29]. Immune aging in LUAD, therefore, more effectively captures the shift toward reduced T-cell competence and increased myeloid activation, features that have been linked to adverse prognosis. In contrast, the more uniform and less immunologically plastic landscape of LUSC may limit the prognostic value of immune aging [30]. These differences may explain why TME immune aging emerged as a stronger prognostic marker solely in LUAD.

Immunosenescence refers to the progressive, age-associated deterioration and reconfiguration of immune function. It encompasses a decline in naïve T-cell generation, reduced proliferative and cytotoxic capacity, chronic low-grade inflammation, and expansion of terminally differentiated or exhausted lymphocyte subsets [6,7,13,31,32]. However, this process does not completely correlate with chronological aging [7]. Especially within TME, persistent tumor antigen stimulation, metabolic stress, and suppressive cues originating from malignant and stromal compartments make heterogeneity in immune aging even among similar age groups [13,33]. Within this context, persistent antigen exposure and metabolic constraints can drive T cells into dysfunctional states such as exhaustion and senescence, characterized by impaired cytotoxic function despite their presence. These processes may contribute to ineffective antitumor immunity within the TME [34]. Meanwhile, it has also been reported that the aging TME is characterized by increased neutrophil infiltration [35], which was similarly observed in our study. Neutrophils within the TME may exhibit functional heterogeneity, with both pro-tumorigenic and anti-tumor roles depending on the local context. Tumor-associated neutrophils may promote tumor progression through immunosuppression and angiogenesis [36], while certain subsets may also contribute to antitumor immune responses [37]. This drives a differential transcriptional landscape characterized by diminished adaptive immune competence and heightened myeloid-inflammatory activity. In this study, higher levels of intratumoral immune aging were strongly associated with inferior survival, particularly in LUAD, suggesting that immunosenescence may actively reshape antitumor immunity and associate with tumor progression. Therefore, it is warranted to keep focusing on tumor immune aging as a distinct and biologically meaningful dimension of LUAD pathophysiology, with potential implications for prognostic stratification and therapeutic response, including immunotherapy.

### Limitations

Our study has several limitations. First, although external validation was performed using two independent cohorts, these datasets were retrospective in nature, and prospective or multi-center validation was not available. Therefore, the generalizability of our findings remains to be confirmed in more diverse and clinically representative populations. Second, as discussed above, the IAS-121 signature was originally developed from peripheral blood transcriptomes; therefore, its application to tumor RNA-seq requires cautious interpretation. It may reflect a tumor-adapted immune aging–like program rather than systemic immune aging. Further studies with paired blood–tumor data are needed to validate this approach. Third, other important clinical confounders—such as detailed treatment information, PD-L1 status, comorbidity burden, and broader genomic alterations—were unavailable in TCGA and could not be incorporated into the models. Fourth, because the cohorts used in this study (TCGA and GSE68465) predominantly consisted of early-stage disease, our findings may not fully represent the biology of advanced LUAD. Given that LUAD is the most common subtype of non-small cell lung cancer worldwide and that the global population is aging, the concept of immune aging within the TME may have broad relevance across diverse populations and warrants further investigation in future studies.

## 5. Conclusions

This hypothesis-generating study shows that immune aging within TME is associated with distinct immunologic features and poorer survival in LUAD. By applying a validated immune-aging gene signature to tumor transcriptomes, we showed that intra-tumoral immune aging reflects a shift toward immunosuppressive myeloid activation and diminished adaptive immune competence. These findings highlight tumor immune aging as a biologically meaningful dimension of LUAD heterogeneity, with potential relevance for prognostic stratification. Future studies warrant focusing on integrating tissue- and blood-based immune aging metrics to better define systemic and intratumoral immune aging relationships, as well as on validating these findings in prospective cohorts.

## Figures and Tables

**Table 1:** Characteristics of patients according to IAS-121 tertile group.

	Lowest (n = 173)	Highest (n = 173)	*p*
**Age (SD)**	67.3 (8.9)	62.6 (10.7)	0.001
**Gender, n (%)**			0.031
Female	103 (59.5)	82 (47.4)	
Male	70 (40.5)	91 (52.6)	
**AJCC stage, n (%)**			0.058
1 & 2	144 (83.2)	125 (76.5)	
3	21 (12.1)	34 (18.0)	
4	5 (2.9)	12 (4.3)	
**Smoking, n (%)**			0.009
Never	29 (16.8)	18 (10.4)	
Former	109 (63.0)	95 (54.9)	
Current	30 (17.3)	56 (32.4)	
**EGFR, n (%)**			0.628
Mutated	20 (11.6)	24 (8.6)	
Wild type	153 (88.4)	149 (91.4)	

SD = standard deviation; AJCC = American Joint Committee on Cancer; IAS = immune aging score; EGFR = epidermal growth factor receptor.

**Table 2:** Cox proportional hazards models comparing the lowest versus the highest tertile of immune aging score-121 and overall survival in lung adenocarcinoma.

	TCGA		GSE68465 Plus GSE50081	
	HR (95% CI)	*p*	HR (95% CI)	*p*
**Crude**	1.86 (1.33–2.62)	<0.001	1.68 (1.19–2.39)	0.004
**Adjusted**	1.87 (1.20–2.92)	0.006	1.57 (1.02–2.43)	0.007

Models adjusted for age, sex, tumor stage, smoking status, and EGFR mutation status for TCGA dataset and age, sex, tumor stage for GSE68465 plus GSE50081 cohorts. HR = hazard ratio; CI = confidence level.

## Data Availability

This study is based upon data generated by the TCGA Research Network, which can be found at https://www.cancer.gov/tcga, and Gene Expression Omnibus, which can be found at https://www.ncbi.nlm.nih.gov/geo/query/acc.cgi?acc=GSE68465.

## Supplementary Materials

The following supporting information can be downloaded at: https://www.mdpi.com/article/10.3390/cancers18091343/s1, Figure S1: Restricted cubic spline curve for the association between IAS-121 and overall survival in patients with TCGA-LUAD; Figure S2: Overall survival according to immune aging score-121 quartiles in TCGA and External Validation Cohorts; Figure S3: Overall survival according to immune aging score-121 median cut-off in TCGA and External Validation Cohorts; Figure S4: Forest plot of subgroup analyses; Figure S5: Heatmap of immune aging-related 121-gene expression in LUAD.

## Abbreviations

CI	Confidence Interval
GDC	Genomic Data Commons
HR	Hazard Ratio
IAS-121	121-Gene Immune Aging Score
LUAD	Lung Adenocarcinoma
TME	Tumor Microenvironment
TCGA	The Cancer Genome Atlas
