# Supplementary material for: Immune Aging Within the Tumor Microenvironment Predicts Survival in Lung Adenocarcinoma
Source: Cancers (Basel). 2026 Apr 23;18(9):1343. doi: 10.3390/cancers18091343 (PMC13162746; doi:10.3390/cancers18091343)
Supplement: Supplementary file 1 [file cancers-18-01343-s001.zip › cancers-4240031-supplementary.pdf]

Supplementary Materials

Immune Aging Within the Tumor Microenvironment Predicts Survival in Non–Small Cell Lung Cancer

Table of Contents

(1) Figure S1

(2) Figure S2

(3) Figure S3

(3) Figure S4

(4) Figure S5

Figure S1. Restricted cubic spline curve for the association between IAS-121 and overall survival in patients with TCGA-LUAD.

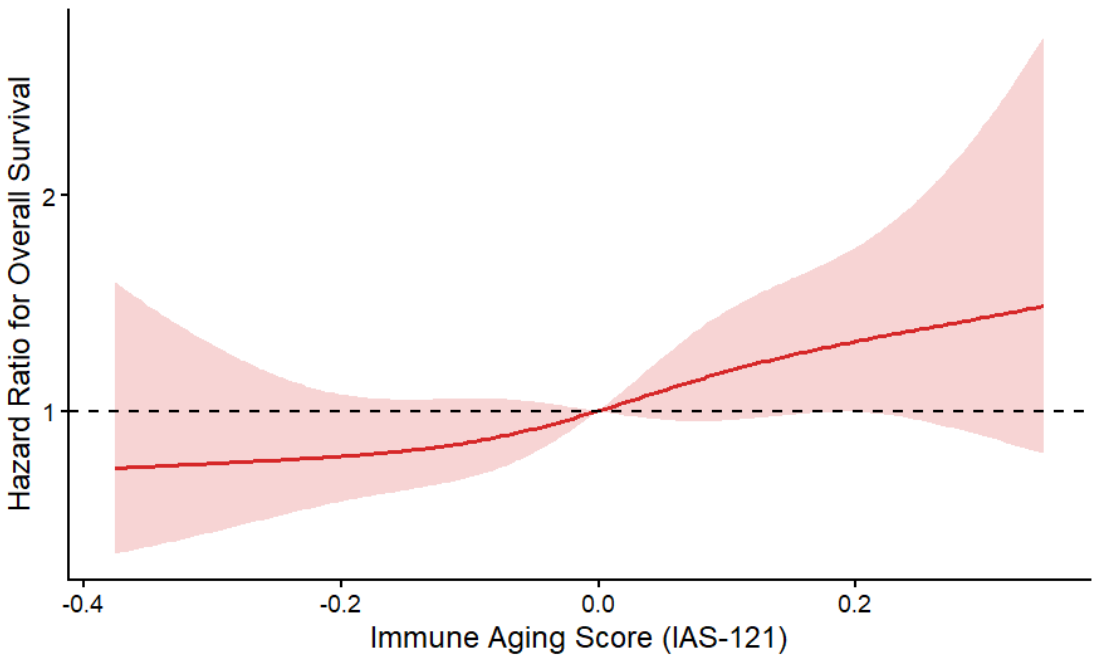

Figure S2. Overall survival according to immune aging score-121 quartiles in TCGA and external validation cohorts.

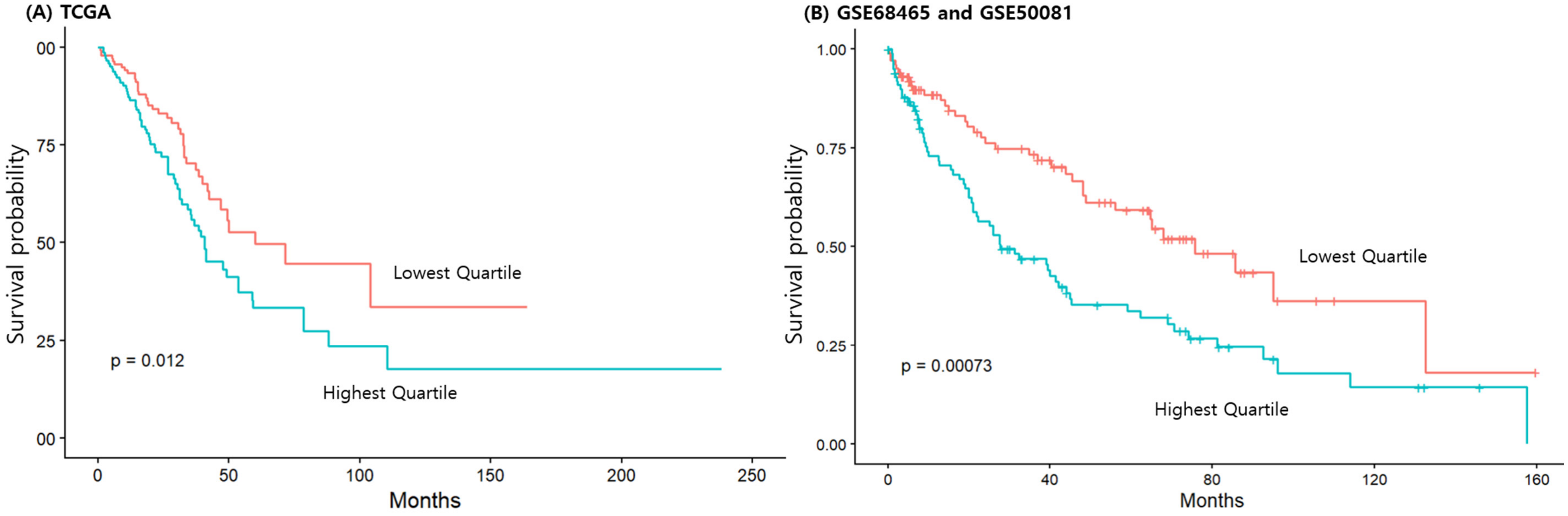

Figure S3. Overall survival according to immune aging score-121 median cut-off in TCGA and external validation cohorts.

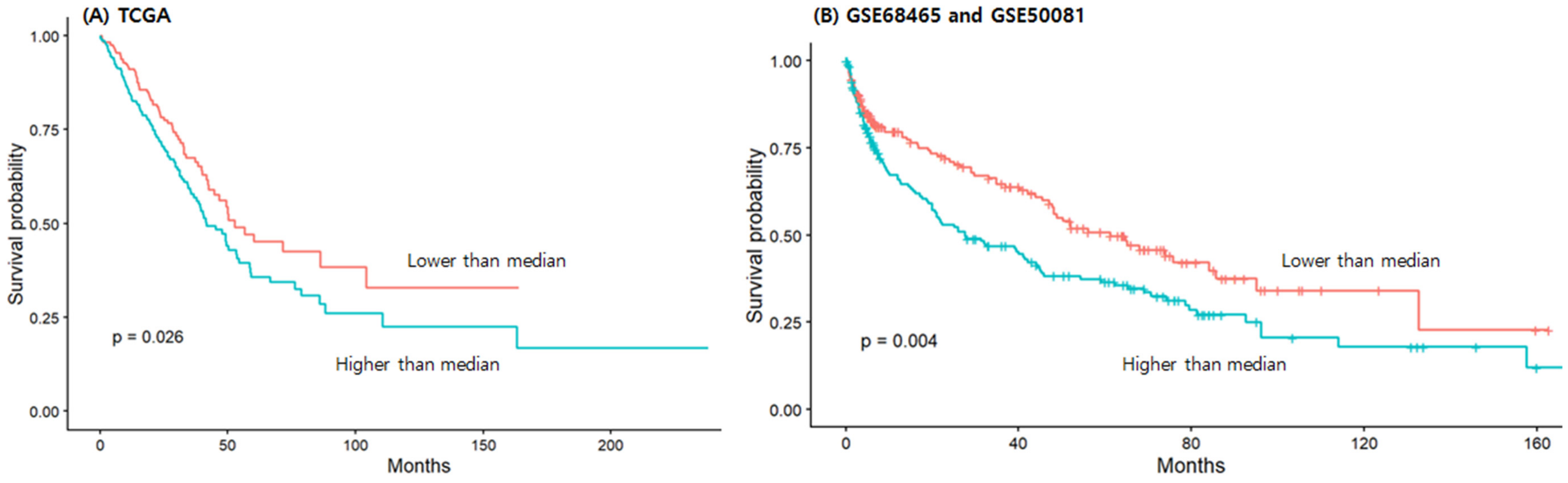

Figure S4. Forest plot of subgroup analyses.

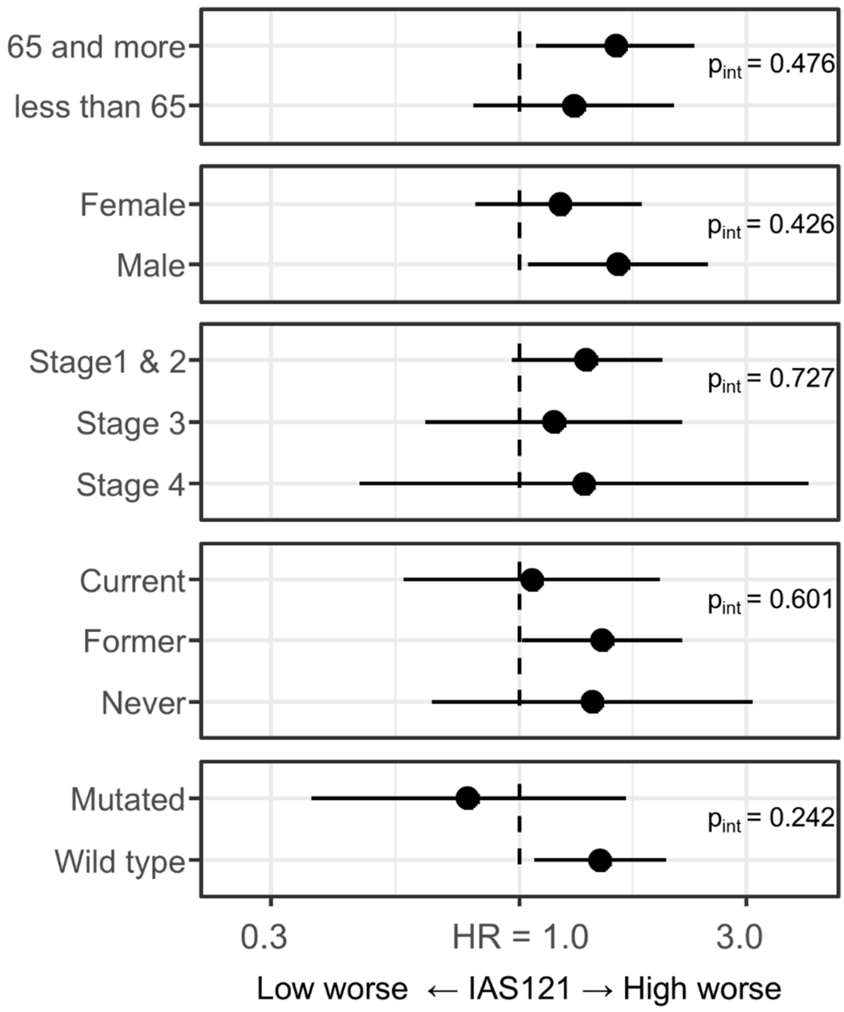

Figure S5. Heatmap of immune aging-related 121-gene expression in LUAD.

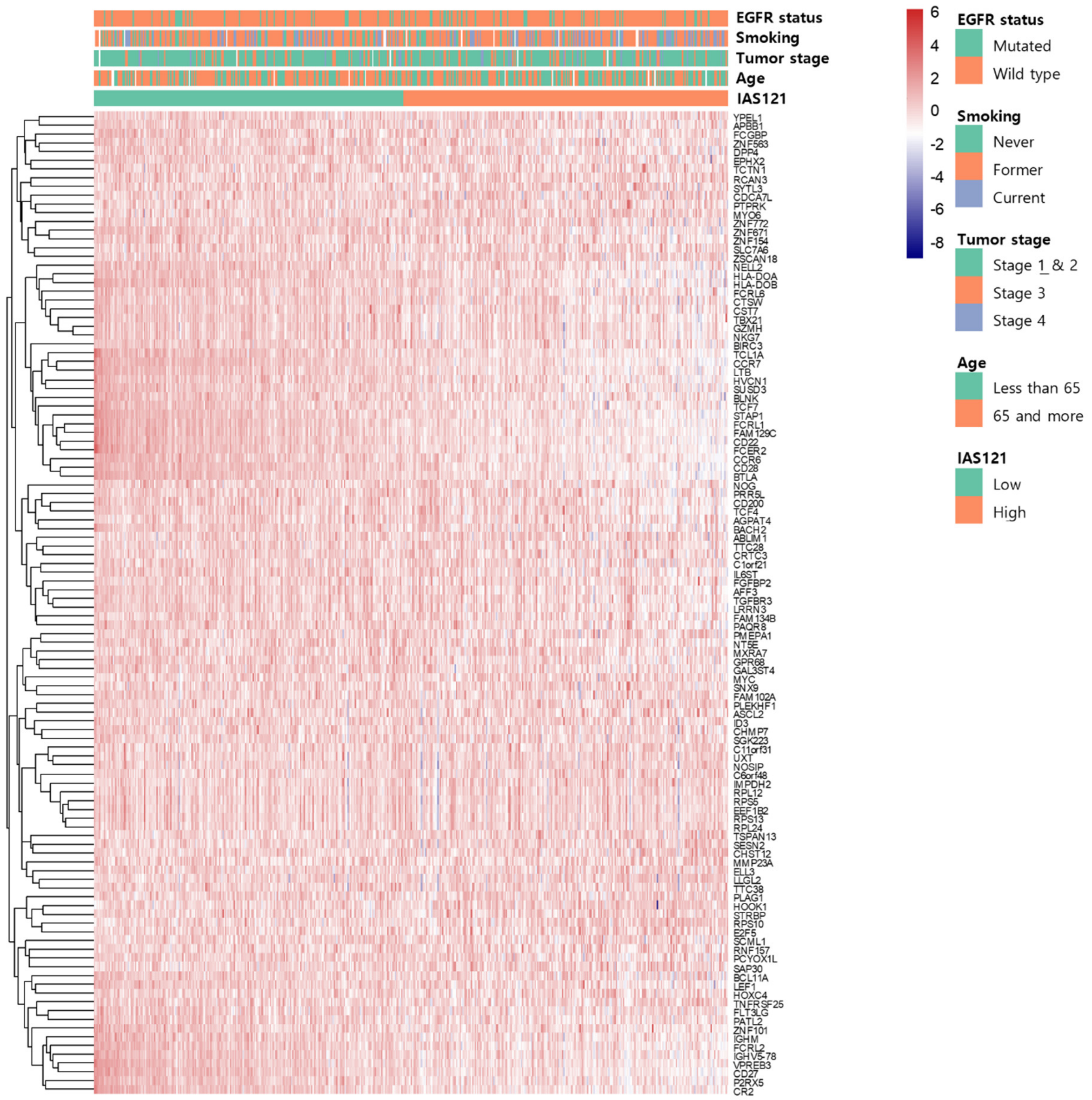

IAS = immune aging score; LUAD = lung adenocarcinoma.
